# Perspectives of primary care healthcare professionals on interventions aimed at reducing the environmental impact of medicines: a mixed-methods systematic review protocol

**DOI:** 10.12688/hrbopenres.14309.1

**Published:** 2026-02-20

**Authors:** Laura Culkin, Helena Cardon, Katja Taxis, Koen Boussery, David Mockler, Cristín Ryan

**Affiliations:** 1 School of Pharmacy and Pharmaceutical Sciences, Trinity College Dublin, Dublin, Leinster, Ireland; 2 Pharmaceutical Care Unit, Faculty of Pharmaceutical Sciences, Ghent University, Ghent, Belgium; 3 Unit of PharmacoTherapy, -Epidemiology & -Economics, Groningen Research Institute of Pharmacy (GRIP), University of Groningen, Antonius Deusinglaan, 1, 9713 AV Groningen, The Netherlands; 4 John Stearne Medical Library, Trinity Centre for Health Sciences, St James's Hospital, Dublin 8, Leinster, Ireland

**Keywords:** Primary care, Healthcare professionals, Perspectives, Interventions, Environmental impact, Medicines

## Abstract

**Background:**

Medicines can affect the environment throughout their lifecycle; from manufacturing to use and disposal. Medicines can enter the environment through various sources, some controllable and others not. Excretion is the primary source of pharmaceutical pollution; however, secondary sources also include improper disposal and effluent discharge. This pharmaceutical pollution can harm both human and animal health. As most prescribing occurs in primary care, it is a key setting for interventions aimed at reducing the environmental impact of medicines. The perceptions of healthcare professionals regarding these efforts remain unclear.

**Aim:**

This systematic review aims to explore the perspectives of healthcare professionals working in primary care regarding interventions designed to reduce the environmental impact of medicines.

**Methods:**

Systematic searches of MEDLINE, Embase, Web of Science, and CINAHL will be undertaken. Studies using qualitative, quantitative, or mixed-methods designs will be included if they explore the perspectives of primary care healthcare professionals on interventions to reduce the environmental impact of medicines. Title and abstract screening, followed by full-text review, will be performed independently and in duplicate. Study quality will be appraised independently by two reviewers using the appropriate Joanna Briggs Institute (JBI) critical appraisal tool. A data extraction tool will be developed, piloted, and applied to collect relevant information, with extraction conducted independently and in duplicate. Quantitative data will be qualitised and integrated with qualitative evidence as part of a convergent integrated synthesis, with findings presented in the form of a narrative synthesis.

**Conclusion:**

This review will help inform the design and implementation of future environmental interventions in primary care. By understanding healthcare professionals’ perspectives on existing or proposed interventions, future strategies can be tailored to improve their acceptability and feasibility.

**Protocol registration:**

This protocol has been registered on PROSPERO (CRD420251247490). Any amendments to this protocol will be documented and uploaded as revision notes on all platforms where the protocol is published.

## Introduction

Environmental pollution with active pharmaceutical ingredients has been recognised since the 1970s and is expected to become an increasing global concern as global life expectancy rises—predicted to increase by 4.5 years between 2022 and 2050—alongside growing pharmaceutical consumption. ^1– 5^ The environmental impact of medicines spans their entire life cycle, from manufacture and use to disposal. ^6^ Globally, healthcare is estimated to contribute 4%–5% of total greenhouse gas emissions, with pharmaceuticals estimated to account for 13%–55% of this footprint. ^7, 8^ Pressurised metered-dose inhalers (pMDIs) are the single largest contributor to medicines-related carbon emissions within the NHS. ^9^ However, despite their substantial carbon footprint, pMDI use remains high. In Ireland, the proportion of pMDIs among all inhalers increased from 49.2% in 2020 to 57.9% in 2021, before slightly decreasing to 54.2% in 2022. ^10^

The environmental fate of medicines does not end with consumption. For some widely used drugs, the majority of an orally administered dose is excreted as active substances. ^11^ Improper disposal of medicines, industrial and hospital effluent, septic tank leakage, and agricultural run-off contribute to the presence of pharmaceuticals in the environment. ^12^ Combined with their high chemical stability, and the limited effectiveness of conventional water treatment methods, these factors lead to the pharmaceutical pollution of surface waters. ^12, 13^

The ecotoxicological risks of pharmaceutical contamination to non-target species have been well established. Diclofenac, for example, causes tissue damage in rainbow trout and impairs growth and hatching in medaka and zebrafish. ^14– 17^ Oestrogens in aquatic environments have been linked to feminisation and intersexuality in male fish, impaired reproduction, and changes in mating behaviour. ^18– 20^ Beyond ecological harm, pharmaceutical pollution poses a threat to human health. A major concern is the increase in the prevalence of bacteria carrying antibiotic resistance genes, attributed to antibiotic pollution. ^21^ Since the 1940s, the prevalence of antibiotic resistance genes in soils in the Netherlands has increased across all classes of antibiotics. ^22^

Healthcare professionals have an important role in mitigating the environmental impact of medicines, and this has been recognised at a global level. In September 2023, the International Pharmaceutical Federation (FIP) issued a *Statement of Policy: Environmental sustainability within pharmacy*, acknowledging the environmental impact of medicines on both climate change and pollution, and called on all pharmacy sectors to mitigate these impacts. ^23^ Similarly, the World Medical Association’s International Code of Medical Ethics (2022) states that “The physician should strive to practise medicine in ways that are environmentally sustainable with a view to minimising environmental health risks to current and future generations”. ^24^

The Pharmaceutical Group of the European Union (PGEU) has highlighted the role of pharmacists in mitigating the environmental risks posed by medicines, including only dispensing necessary quantities, implementing environmentally safe medicine disposal practices within their pharmacies, and facilitating the safe disposal of medicines by the public. ^25^ Evidence suggests that healthcare professionals can directly influence environmentally sustainable practices among the public. A survey performed by Vellinga *et al.* found that 72% of respondents who had previously disposed of medicines had done so incorrectly. However, among those who had received advice from a healthcare professional on disposal practices, this figure was only 25%. ^26^

Primary care is the principal setting in which healthcare is delivered in Europe, with the majority of prescribing and management of chronic diseases occurring in this setting. ^27, 28^ As healthcare professionals in primary care are central to the prescribing, dispensing, and management of medicines, understanding their perspectives on interventions aimed at reducing the environmental impact of medicines is essential, as these perspectives directly influence the acceptability, implementation, and sustainability of these interventions. By capturing perspectives from international studies of healthcare professionals involved in prescribing, dispensing, and medicines management in primary care, this review will inform the development and implementation of future environmentally sustainable initiatives.

### Rationale for the research

To date, individual studies have investigated environmental initiatives and explored healthcare professionals’ knowledge, awareness, and engagement with sustainability practices. However, these findings remain fragmented and, as far as the authors are aware, no systematic review has identified, appraised, and synthesised evidence to understand the perspectives of healthcare professionals in primary care regarding interventions aimed at reducing the environmental impact of medicines worldwide. This protocol outlines a mixed-methods systematic review designed to address this gap.

## Aims and objectives

The overall aim of this systematic review is to synthesise the available evidence on the perspectives of healthcare professionals working in primary care regarding interventions to reduce the environmental impact of medicines.

The objectives of this systematic review are to:

• Identify studies that describe primary care interventions specifically intended to reduce the environmental impact of medicines.
• Explore healthcare professionals’ perspectives on these interventions, including views, beliefs, attitudes, knowledge, practices, and experiences.
• Compare and contrast the perspectives of primary care healthcare professionals across countries and/or healthcare sectors, where feasible.
• Integrate and synthesise qualitative and quantitative evidence to provide a comprehensive understanding of healthcare professionals’ perspectives on interventions to reduce the environmental impact of medicines in primary care.

## Methods

This protocol was developed in line with the Preferred Reporting Items for Systematic Reviews and Meta-Analyses Protocols (PRISMA-P) guidelines. ^29^ It is also registered on PROSPERO, an international register of systematic reviews.

A mixed-methods systematic review was chosen to facilitate the integration of quantitative, qualitative, and mixed-methods evidence, providing a more comprehensive understanding of the topic than any single method alone. ^30^ By considering diverse evidence types, the authors aim to maximise the findings and capture different aspects and perspectives.

### Inclusion criteria

The PICo (Population, Phenomenon of Interest and Context) tool was used to define the research question, synthesise the search strategy, and refine the inclusion and exclusion criteria. This tool is recommended by the Joanna Briggs Institute (JBI) for mixed-methods systematic reviews (MMSRs), which follow a convergent integrated approach. ^31^

*Population:* The population of interest is healthcare professionals employed in primary care who are directly involved in medicine-related activities for humans, such as prescribing, dispensing, counselling, or administration. This includes community pharmacy staff, general practice staff, dentists, and primary care nurses. Primary healthcare professionals without a medicine-related role (e.g., dietitians, physiotherapists, speech and language therapists, occupational therapists), those working outside primary care, or those involved in medicine-related activities for animals (e.g., veterinary professionals, veterinary nurses) will be excluded. Studies including both eligible and ineligible populations will be included if data for the eligible population can be distinguished.

*Phenomenon of Interest:* This review will consider studies describing the perspectives of healthcare professionals employed in primary care regarding interventions that specifically aim to reduce the environmental impact of medicines.

For the purpose of this review, “perspectives” refers to views, beliefs, attitudes, knowledge, practices, and experiences regarding these interventions.

Interventions may be enacted (implemented in primary care) or proposed (hypothetical, discussed, or planned) but must explicitly aim to reduce the environmental impact of medicines used by humans. Studies describing interventions with only incidental or unintentional benefits (e.g., deprescribing to reduce polypharmacy) will be excluded, unless an environmental rationale was a stated aim or motivation. Studies describing multiple interventions will be included if at least one intervention was implemented to reduce the environmental impact of medicines, and the data can be delineated.

Examples of eligible interventions include, but are not limited to:

- Pharmacy take-back schemes to prevent inappropriate disposal of medicines.
- Prescribing dry powder inhalers (DPIs) instead of pressurised metered-dose inhalers (pMDIs).
- Medication reviews that state a reduction in environmental impact as an objective.
- Recommending medicines with a lower environmental impact.

*Context:* The context of this review is any primary care setting worldwide in which healthcare professionals involved in medicine use are employed. Initiatives performed outside of primary care will be excluded. This broad context will facilitate a comprehensive understanding of healthcare professionals’ perspectives across different primary care settings and countries. The authors acknowledge that legislation and the roles of healthcare professionals may differ between countries.

### Search strategy

The search strategy was developed in consultation with the subject librarian, Mr David Mockler, and will be applied to CINAHL, EMBASE, Medline, and Web of Science. The full search strategies are available at https://doi.org/10.17605/OSF.IO/5QADP. ^32^

An initial trial search of EMBASE was performed to identify keywords and relevant index terms, which were then iteratively refined to develop the search strategy. The search strategy was then adapted for each database. Search terms focus on keywords and controlled vocabulary pertaining to ‘primary care’, ‘interventions’, ‘perspectives’, ‘healthcare professionals’, ‘environmental impact’, and ‘medicines’.

Searches will not be limited by year or by language. However, non-empirical studies (such as commentaries, opinion pieces, conference abstracts) will be excluded. Additional relevant literature will be identified through forward and backward citation searching of included papers.

### Study selection

Two authors (LC, HC) will independently assess studies against the inclusion criteria. Draft inclusion and exclusion criteria are presented in Table 1 *.* Following searches of all databases, identified citations will be imported into Covidence and duplicate records will be removed.

**Table 1.** Details of inclusion/exclusion criteria.

Criterion	Inclusion	Exclusion
Population	- Healthcare professionals employed in primary care who have a medicine-related role, e.g., community pharmacy staff, general practice staff, dentists, and primary care nurses.	Perspectives of the following populations will not be considered: - Non-healthcare professionals. - Healthcare professionals working outside of primary care. - Healthcare professionals without a medicine-related role (e.g., dietitians, physiotherapists).
Phenomenon of interest	- Interventions may be proposed or enacted but must have the specific intention of reducing the environmental impact of medicines. - Perspectives (knowledge, views, beliefs, attitudes, practices, and experiences) of healthcare professionals regarding these interventions.	- Interventions which do not reduce the environmental impact of medicines. - Interventions which unintentionally reduce the environmental impact of medicines. - Studies that do not describe healthcare professionals’ perspectives on interventions.
Context	- Primary healthcare settings globally will be considered.	- Non-healthcare settings. - Healthcare settings outside of primary care.
Language	- Studies will not be limited by language.	
Year	- Any year.	
Study type	- Qualitative, quantitative, or mixed-method empirical studies.	- Non-empirical studies (e.g., commentaries, conference abstracts, opinion pieces).

To ensure consistency in application of the inclusion and exclusion criteria, LC and HC will pilot test the screening process using a subset of abstracts. LC and HC will then independently and in duplicate screen titles and abstracts. Any uncertainty or disagreement at this stage will result in the study progressing to full-text review.

Full texts of potentially eligible studies will be retrieved and independently assessed by LC and HC. Disagreements at the full-text stage will be resolved through discussion, with consultation from a third reviewer if required. Reasons for exclusion at the full-text review stage will be documented.

The study selection process will be presented using a Preferred Reporting Items for Systematic Reviews and Meta-Analyses (PRISMA 2020) flowchart.

### Data extraction

A modified version of the standardised JBI Mixed Methods Data Extraction Form will be used by two independent reviewers (LC, HC) to extract data from included studies. ^33^ A draft extraction form is presented in Table 2. The data extraction tool will be piloted on at least five included papers and updated iteratively until a consensus is reached between LC and HC. LC and HC will independently extract data from each of the included studies, with extracted data cross-checked. Any discrepancies will be resolved through discussion, or, where consensus cannot be reached, by consulting a third reviewer.

**Table 2.** Data extraction form.

Title	
Author(s)	
Publication year	
Country/geographical location	
Primary care setting	
Study aim(s)	
Study design/methodology	
Healthcare professional type(s)	
Sample size	
Intervention(s) description	
Intervention status (enacted/proposed)	
Perspectives identified	
Limitations	

Information extracted will include: (a) title of paper, (b) author(s), (c) publication year, (d) country/geographical location, (e) primary care setting, (f ) study aim(s), (g) study design/methodology, (h) healthcare professional type(s), (i) sample size, (j) intervention(s) description, (k) intervention status (enacted/proposed), (l) perspectives identified, (m) limitations. If required, authors of included studies will be contacted for missing information or if clarification is needed. Any updates to the data extraction tool will be noted in the systematic review.

### Assessment of methodological quality

The quality of included studies will be appraised for methodological quality independently and in duplicate by LC and HC using the appropriate JBI critical appraisal tools. ^34^ The relevant JBI tool will be selected based on the study design. For mixed-methods studies, the qualitative and quantitative components will be appraised separately using the most appropriate tool. Studies will not be excluded on the basis of low methodological quality; however, methodological limitations and potential biases will be considered when interpreting the findings.

### Assessment of confidence in findings

As per JBI guidance, assessment of confidence in findings using ConQual or GRADE approaches is not recommended. ^35^ As an alternative, identified themes will be compared with the primary studies through repeated examination of the extracted data to ensure accurate representation of findings.

### Data transformation

Quantitative data will be transformed into qualitative data to facilitate integration with qualitative evidence as part of a convergent integrated synthesis. This approach is consistent with JBI guidance, which recommends codifying quantitative data instead of adding numerical values to qualitative data, as the latter is more prone to error. ^31^ The method for data qualitisation follows guidance from the JBI MMSR Methodology Group. ^36^ In accordance with this guidance, where numeric data are presented alongside an accurate narrative representation, these data will be extracted verbatim. If a verbatim extract does not address the review question, LC and HC will only add contextual information explicitly reported elsewhere in the study. If a study reports quantitative results without any accompanying narrative, LC and HC will construct a narrative representation of the results, following the style and format used by the primary authors to narrate other results.

### Data synthesis

The review question may be answered by both qualitative data and quantitative data and will follow a convergent integrated approach. Data synthesis will be performed following the JBI methodology for mixed-methods systematic reviews. ^31^ After transforming quantitative data into qualitative form as described above, these textual descriptions will be combined with findings extracted from qualitative studies. In line with JBI meta-aggregative principles, this combination of extracted data will undergo repeated, detailed examination to identify categories of meaning. ^31^ Categories will be developed by grouping two or more findings with similar meaning. Findings that cannot be grouped will remain as stand-alone categories. Once categories have been formed, related categories will be combined to form higher-level synthesised findings.

### Data dissemination

Findings will be disseminated through academic channels, including at research conferences and through publication in a relevant, high-impact journal and through promotion using internet platforms.

## Conclusion

This mixed-methods systematic review will synthesise global evidence on the perspectives of healthcare professionals employed in primary care regarding interventions aimed at reducing the environmental impact of medicines. By integrating qualitative and quantitative data, the review will provide a comprehensive understanding of perspectives such as acceptability, feasibility, and perceived barriers and enablers to such interventions in primary care. By understanding the perspectives of healthcare professionals, the findings will help inform the development and implementation of future environmentally sustainable initiatives.

## Ethics and consent

Ethical approval and consent were not required.

## Software availability

Covidence will be used for study screening and selection as well as for removal of duplicates.

## Data Availability

No data are associated with this article. Open Science Framework (OSF): Perspectives of primary care healthcare professionals on interventions aimed at reducing the environmental impact of medicines: a mixed-methods systematic review protocol: Extended data. https://doi.org/10.17605/OSF.IO/5QADP. ^32^ Open Science Framework: PRISMA-P checklist for ‘Perspectives of primary care healthcare professionals on interventions aimed at reducing the environmental impact of medicines: a mixed-methods systematic review protocol’ https://doi.org/10.17605/OSF.IO/5QADP. ^32^ Data are available under the terms of the Creative Commons Zero “No rights reserved” data waiver (CC0 1.0 Public domain dedication).
