# Supplementary material for: Perspectives of primary care healthcare professionals on interventions aimed at reducing the environmental impact of medicines: a mixed-methods systematic review protocol
Source: HRB Open Res. 2026 Feb 20;9:19. [Version 1] doi: 10.12688/hrbopenres.14309.1 (PMC13019037; doi:10.12688/hrbopenres.14309.1)
Supplement: Peer review history

# Reviewer report: Reviewer response for version 1

**DOI:** 10.21956/hrbopenres.15750.r53924

**Published:** 2026-03-25

**Authors:** Helen Twohig

**Affiliations:** 1 Keele University, Keele, UK

This is a protocol for a mixed methods systematic review seeking to synthesise evidence on the perspectives of primary health care professionals on interventions aimed at reducing the environmental impact of medicines. This is an important question as the knowledge generated will inform development and implementation of future interventions.

 The protocol is clearly written and the methods are appropriate, following the latest JBI guidance on conducting this type of review. 

 Some points for the team to consider are:

 Population - exclusion criteria include studies on dieticians and SALTs, the stated reason being that these professionals do not have a medicines related role. However, the use of thickened fluids and sip feeds, which these professions do have a role in influencing, contribute to significant environmental impact / waste and thus this exclusion may benefit from being reconsidered. 

 Phenomenon of interest - interventions which unintentionally reduce environmental impact are excluded. However, many interventions have dual benefits in that they may be implemented to improve clinical care (changing to AIR / MART regimes in asthma, reducing polypharmacy to reduce adverse clinical events) but will also have an environmental benefit. Clinician perspectives on these interventions may include thoughts on their environmental impact even if this isn't a stated aim of the intervention. 

 Background section - this is a minor point but I found the background argument rather disjointed and it would benefit from structural revision to make it more coherant when writing up the review.

Is the study design appropriate for the research question?

Yes

Is the rationale for, and objectives of, the study clearly described?

Yes

Are sufficient details of the methods provided to allow replication by others?

Yes

Are the datasets clearly presented in a useable and accessible format?

Not applicable

Reviewer Expertise:

Primary care research, environmental sustainability of healthcare

I confirm that I have read this submission and believe that I have an appropriate level of expertise to confirm that it is of an acceptable scientific standard.

# Reviewer report: Reviewer response for version 1

**DOI:** 10.21956/hrbopenres.15750.r53752

**Published:** 2026-02-25

**Authors:** Amy Booth

**Affiliations:** 1 University of Oxford, Oxford, UK

This article outlines a systematic review protocol intended to examine primary care healthcare professionals’ (HCPs) perspectives on interventions aimed at reducing the environmental impact of medicines. The topic is important and justified, given increasing recognition of medicines’ environmental impact and the role of HCPs in mitigating this impact. Overall, the rationale for the review is clearly described and well justified. The proposed study design is appropriate for the research questions, and the methods are clearly outlined and sufficiently detailed to allow replication. 

 The comments below are intended as constructive suggestions to strengthen the article before the review is undertaken and when the completed manuscript is prepared for indexing.

 1. **Background**

 The background section is generally well-written and appropriate for a protocol. However, when preparing the completed review for publication, the narrative flow could be improved. For example, at present, the text moves between different types of environmental impacts (e.g., pharmaceuticals in the environment, climate change) and different mechanisms of impact (e.g., various lifecycle stages, prescribing practices, disposal) which makes it tricky to follow.

**2. Review objectives**

 The protocol currently proposes to identify studies describing interventions to reduce the environmental impact of medicines (Objective 1) and to explore HCP perspectives on these interventions (Objective 2). While each objective is individually reasonable, there is potential that studies that describe interventions (Objective 1) may not assess HCP perspectives, and that studies that explore HCP perspectives (Objective 2) may not evaluate or describe specific interventions. This raises a potential scenario where the review may inadvertently require two distinct evidence bases (i.e., one that assesses interventions and one that assesses HCP perspectives) in order to meet the specified objectives. The authors may wish to either reframe the review objectives to focus specifically on HCP perspectives on interventions to reduce the environmental impact of medicines, rather than first identifying interventions and then separately exploring perspectives or clearly define a staged approach (e.g., first mapping interventions, then synthesising perspective data only from studies that include such data). If not, it would be worth thinking about how you will handle studies that describe interventions without assessing perspectives.

 3. **Definition of the population**

 Please note that in some healthcare systems, prescribing rights extend to physiotherapists, speech and language therapists, advanced nurse practitioners, other allied health professionals etc. So it might be worth revisiting your exclusion of some of these professions.

 Otherwise good luck with conducting the review and I look forward to reading the results!

Is the study design appropriate for the research question?

Yes

Is the rationale for, and objectives of, the study clearly described?

Yes

Are sufficient details of the methods provided to allow replication by others?

Yes

Are the datasets clearly presented in a useable and accessible format?

Not applicable

Reviewer Expertise:

Sustainable health care; pharmaceutical supply chain; sustainable medicines; climate change; pharmaceuticals in the environment

I confirm that I have read this submission and believe that I have an appropriate level of expertise to confirm that it is of an acceptable scientific standard.
